# Enhanced Mucosal Antibody Production and Protection against Respiratory Infections Following an Orally Administered Bacterial Extract

**DOI:** 10.3389/fmed.2014.00041

**Published:** 2014-10-30

**Authors:** Christian Pasquali, Olawale Salami, Manisha Taneja, Eva S. Gollwitzer, Aurelien Trompette, Céline Pattaroni, Koshika Yadava, Jacques Bauer, Benjamin J. Marsland

**Affiliations:** ^1^OM Pharma SA Geneva, Geneva, Switzerland; ^2^Faculty of Biology and Medicine, University of Lausanne, Service de Pneumologie, CHUV, Lausanne, Switzerland

**Keywords:** influenza, lung, super-infection

## Abstract

Secondary bacterial infections following influenza infection are a pressing problem facing respiratory medicine. Although antibiotic treatment has been highly successful over recent decades, fatalities due to secondary bacterial infections remain one of the leading causes of death associated with influenza. We have assessed whether administration of a bacterial extract alone is sufficient to potentiate immune responses and protect against primary infection with influenza, and secondary infections with either *Streptococcus pneumoniae* or *Klebsiella pneumoniae* in mice. We show that oral administration with the bacterial extract, OM-85, leads to a maturation of dendritic cells and B-cells characterized by increases in MHC II, CD86, and CD40, and a reduction in ICOSL. Improved immune responsiveness against influenza virus reduced the threshold of susceptibility to secondary bacterial infections, and thus protected the mice. The protection was associated with enhanced polyclonal B-cell activation and release of antibodies that were effective at neutralizing the virus. Taken together, these data show that oral administration of bacterial extracts provides sufficient mucosal immune stimulation to protect mice against a respiratory tract viral infection and associated sequelae.

## Introduction

Recurrent respiratory tract infections (RTIs) are a leading cause of morbidity and mortality, with therapeutic options largely limited to traditional antibiotic treatments (1). The cause of susceptibility to RTIs is varied, but overall reflects an inability of the immune system to prevent productive infections (2). Thus, treatment regimes that enhance the immune system’s effectiveness are a valid and rational approach.

The immune system is immature at birth, and following microbial colonization and host-microbe interactions, it matures and develops the capacity to effectively control infections (3). Studies utilizing axenic (germ-free) mice, which harbor no microbes, have shown that in the absence of microbial colonization, there is lymphopenia, drastically reduced levels of mucosal IgA, and impaired epithelial barrier integrity (4). Recent research highlights the importance of host–microbe interactions in both health and disease, and these data support the concept of modulating the host microbiota by utilizing prebiotics or probiotics for disease prevention (5). An alternative approach is the direct administration of purified microbial components or bacterial extracts that provide maturation signals to the immune system (6–9). Indeed, both clinical and experimental studies have shown that administration of bacterial extracts can be efficacious in numerous disease settings such as chronic obstructive pulmonary disease, recurrent respiratory and urinary tract infections of bacterial or viral origins, wheezing lower respiratory illness (WLRI), and subsequent asthma (10, 11).

Experimental studies support the concept that exposure to bacterial components may influence the response to a variety of pathogens. Mice pretreated with an aerosolized *Haemophilus influenzae* lysate were protected against respiratory infection with a variety of pathogenic bacteria and fungi, including *Streptococcus pneumoniae, Klebsiella pneumoniae, Pseudomonas aeruginosa, Bacillus anthracis*, and *Aspergillus fumigatus* (12). The same treatment also protected mice against nebulized influenza virus (13). In the 90s, and more recently, clinical studies demonstrated that treatment with a bacterial extract, OM-85 (marketed as Broncho-Vaxom, Broncho-Munal, Ommunal, Paxoral, Vaxoral), was effective against different RTIs (14–17). Efficacy in reducing morbidity was also shown with OM-85 in children affected by recurrent RTIs (18) and significantly reduced the rate and duration of virus-induced WLRI in preschool children with acute RTI (19). As with the general influence of microbial colonization, the immune stimulatory pathways elicited by bacterial extracts are varied. Recent data indicate that TLR4 and TLR2 could be stimulated by OM-85 (20), and an influence upon expansion of T regulatory cells and enhancement of T helper type 1 responses has been reported (21).

In the present investigation, we have assessed the efficacy and mechanism of action of OM-85 in mouse models of respiratory infections. OM-85’s active principle ingredients are composed of soluble extracts of lysates from 21 bacterial strains (5 pathogenic bacterial genera) that are mainly responsible for RTIs [*H. influenzae, Streptococcus* (pneumonia, pyogenes, and sanguinis), *Klebsiella* (*pneumoniae* ssp. *pneumoniae* and *pneumoniae* ssp. *ozaenae*), *Staphylococcus aureus*, and *Moraxella catarrhalis*]. It is utilized in adults and in children as of 12 months of age for immunotherapy, prevention of recurrent infections of the respiratory system and acute and chronic bronchitis, and co-medication in the treatment of acute respiratory infections. We show that treatment of mice with OM-85 acts to enhance innate and adaptive arms of the immune system with consequently improved control of influenza virus infection. As reported previously in influenza vaccination studies (22, 23), such enhanced immunity had highly relevant consequences for secondary infections, where it led to improved outcomes following subsequent challenge by either Gram-positive or Gram-negative bacteria. The mechanism of action underlying the OM-85 mediated protective effect was unique in that it involved the induction of polyclonal mucosal antibody responses that neutralized viral particles, thus blocking the infection. This innate antiviral response was also associated with enhanced adaptive CD8+ T-cell response, indicating that the actions of OM-85 were multifactorial.

## Materials and Methods

### Mice and infections

Female BALB/c mice aged 8 weeks were purchased from Charles River. Influenza virus strain PR8 (A/Puerto Rico8/34, H1N1) was sourced from Virpur (San Diego). Viral infections were performed by intranasal administration of 100 PFU of virus in 50 μl of PBS. *S. pneumoniae* strain D39 glycerol stocks were inoculated into 5 ml of BHI Broth and incubated overnight at 37°C and 5% CO_2_, without shaking. After 24 h, 50 ml of BHI broth was inoculated with 500 μl of overnight stationary culture in a 250 ml flask, and incubated at 37°C, 5% CO_2_, without shaking. The culture was sampled at several time points (5, 6, 7, and 8 h), O.D. 620 nm was determined and CFU/ml controlled by plating bacteria on Mueller Hinton + 5% sheep blood agar plates. Mice were infected intranasally with 50 μl of the bacterial solution containing 1 × 10^5^ CFU. For preparation of *K. pneumoniae*, 5 ml of Nutrient Broth (NB) was inoculated with glycerol stock in a 14 ml round-bottomed tube and incubated at 37°C with shaking. After 24 h, 50 ml of NB was inoculated with 500 μl of the overnight stationary culture in a 250 ml flask followed by incubation at 37°C with shaking. The culture was sampled at several time points to measure O.D. at 620 nm and CFU/ml controlled by plating bacteria on NB plates. Mice were infected with 50 μl of the bacterial solution (endotoxin and DNA free content) containing 10^1^ CFU of bacteria. For *in vivo* experiments, daily gavage of mice with 7.2 mg of OM-85-active principle (corresponding to 320 μL concentrate) or its equivalent without bacterial extract (referred to as “control solution”) was performed during 10 days. Animal experiments were performed in accordance with the Institutional Guidelines and Swiss Federal and Cantonal Laws on Animal Protection.

#### Quantification of bacterial load in blood

Twenty-four hours following infection, blood was sampled and serially diluted followed by plating on LB (Klebsiella) or Mueller Hinton + 5% sheep blood (*Streptococcus*) agar plates and incubation overnight at 37°C. Following 24 hours of incubation, bacterial colonies were counted and CFUs extrapolated.

#### Disease scoring

In all, 1 point for a healthy mouse; 2 points for a mouse showing signs of malaise, including slight piloerection, slightly changed gait, and increased ambulation; 3 points for a mouse showing signs of strong piloerection, constricted abdomen, changed gait, and periods of inactivity; 4 points for a mouse with enhanced characteristics of the previous group, but showing little activity and becoming moribund; and 5 points for a dead mouse.

### Antibodies and flow cytometry

Different combinations of the following antibodies were used for flow cytometry. For analysis of dendritic cell (DC) subsets in the lung, a combination of CD11b PerCP-Cy5.5, CD11c APC-Cy7, B220 FITC, MHC II Alexa Fluor 700, CD40 PE, ICOS-L PE, CD80 PE, or CD86 PE was used. Analysis of B-cells subsets was performed with CD19 PE, CD23 PE-Cy7, CD21 Pacific Blue, IgD FITC, and IgM APC. Influenza-specific CD8+ T-cells were stained with NP-tetramer 366–374. All antibodies were purchased from Biolegend (San Diego) unless indicated. Stained cells were acquired on a BD FACS CANTO or LSRII and analyzed with FlowJo software (Tree Star, Inc.).

### Quantitative real-time PCR

Total RNA was purified from cells obtained from whole lung and trachea of mice using Tri reagent (Sigma Aldrich). Real-time PCR was carried out according to manufacturers’ instructions using the quantifast SYBR green RT-PCR kit (Qiagen). The following primers were used: beta-Actin forward 5′-CCCTGAAGTACCCCATTGAAC-3′ and reverse 5′-CTTTTCACGGTTGGCCTTAG-3′; influenza matrix protein forward 5′-GGA CTG CAG CGT AGA CGC TT-3′, reverse 5′-CAT CCT GTT GTA TAT GAG GCC CAT-3′. Data are represented as the ratio of the *Cq* values from the influenza matrix protein gene to the house-keeping gene, beta-Actin.

#### Analysis of cells from the bronchoalveolar lavage and lungs

BAL was performed by flushing the airways twice with 500 μl of PBS/0.1% BSA. Total cell counts were determined using a Beckmann Coulter Counter. Cells from BAL were spun onto glass slides using a Cytospin 3 (Shandon). Slides were then stained using Diff Quick staining set (Dade Behring, Siemens Healthcare Diagnostics, Deerfield, IL, USA) and differential cell counts were determined microscopically. Percentages of macrophages, neutrophils, eosinophils, and lymphocytes were determined within a total population of 200 cells.

For isolation of cells from the lung, lungs were perfused with PBS and then digested in medium supplemented with 2 mg/ml Collagenase IV (Invitrogen-Gibco). Single cell suspension was obtained by smashing digested lungs through a 70 μM cell strainer (Milian Falcon).

#### *In vitro* activation of splenocytes

Spleens were isolated from BALB/c mice and pooled. Tissue was placed in IMDM media containing collagenase, cut into approximately 2 mm cubes and incubated for 45 min at 37°C with gentle shaking. Following the incubation, the remaining tissue and media was pressed through 70 μm cell strainers and washed with PBS containing 0.2% BSA. Red blood cells were lysed, and cells were counted using a Coulter Counter (IG Instruments, Zurich, Switzerland). Cultures with 1 × 10^6^ cells per well in 48 well plates (Nunc) with a volume of 500 μl media were setup in triplicate. Cells were stimulated with OM-85 at concentrations of 1 mg/ml, 100 μg/ml, 10 μg/ml, 1 μg/ml, or 0 μg/ml.

### Statistical analysis

Student’s *t* test (unpaired, two-tailed) or a one-way ANOVA was used to calculate significance levels between treatment groups, as indicated. Graph generation and statistical analysis were performed using Prism version 5 software (GraphPad, La Jolla, CA, USA). Standard deviation (SD) was used.

## Results

### The bacterial extract, OM-85, elicits an enhanced innate response that controls influenza virus infection

**Figure 1:**
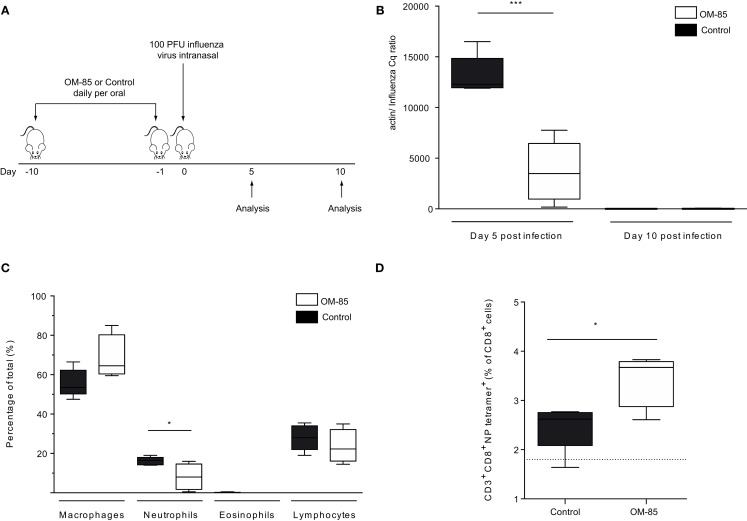
**Oral treatment with OM-85 protects mice against influenza virus infection**. **(A)** BALB/c mice were treated with OM-85 or control solution daily for 10 days. The following day mice were infected with influenza virus. **(B)** The viral load in lung tissue was determined on day 5 and 10 post influenza virus infection. **(C)** Infiltration of inflammatory cells into the airways on day 5 post-infection as determined by standard morphological and cytochemical properties. **(D)** The proportion of influenza-specific NP-tetramer+ cells in the BALF was determined by flow cytometry. Data are representative of 2–5 experiments with 5–10 mice per time point. Error bars represent minimum to maximum value range. Statistical analysis was performed by Student’s *t* test. *p < 0.05; ***p < 0.001.

In order to gain insight into whether and how treatment of mice with OM-85 could enhance antiviral immunity, mice were treated orally with OM-85 for 10 days followed by infection with a sublethal dose of influenza A virus (Figure 1A). In this well-defined infection model, the peak of viral load is on day 5 post-infection and the virus is cleared by day 10 post-infection (24). Mice treated with OM-85 had a lower viral load in the lung tissue on day 5 post-infection as compared to mice treated with the control solution, which had undergone the same manufacturing process as OM-85, but without the bacterial extracts (Figure 1B). On day 10 post-infection, both groups had cleared the virus, as expected, showing that OM-85 treatment did not prolong the infection (Figure 1B). Analysis of the cellular infiltrates into the airways following infection showed that the OM-85 treated mice had a reduced proportion of neutrophils in the airways on day 5 post-infection (Figure 1C). These data corroborated the reduced viral load and indicated that the protective mechanism associated with OM-85 treated mice involved very early control of the infection, and as a consequence of this early control, the neutrophilic response resolved faster. Although the OM-85 mediated protective effect in this case was linked with time points associated with the innate antiviral response, an enhanced adaptive CD8+ T-cell response was noted on day 10 post-infection (Figure 1D). These data suggest that the actions of OM-85 were multifactorial, enhancing both innate and adaptive immune pathways, but the early innate response was likely to be responsible for the efficacy against influenza virus.

### OM-85-mediated enhanced antiviral immunity results in protection against secondary bacterial infections

**Figure 2:**
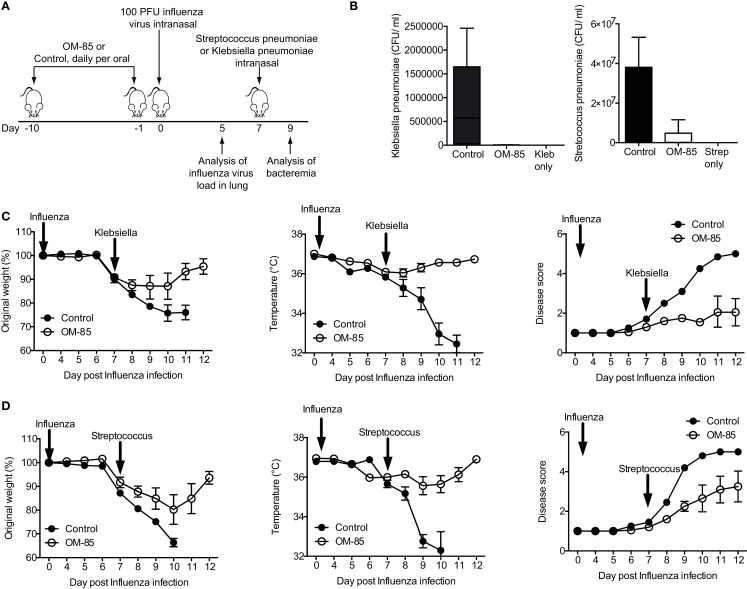
**Oral treatment with OM-85 protects mice against secondary bacterial infections following influenza**. **(A)** Experimental overview. BALB/c mice were treated with OM-85 or control solution daily for 10 days. The following day mice were infected with Influenza virus and 7 days later mice were infected with the indicated bacteria. **(B)** Bacteremia at 24 h following infection of the indicated bacteria. **(C)** Weight, temperature, and disease score of mice control or OM-85 treated mice following influenza infection and *Klebsiella pneumoniae*. **(D)** Weight, temperature, and disease score of control or OM-85 treated mice following influenza infection and *Streptococcus pneumoniae*. Data are representative of 3 experiments with 5–10 mice per time point. Error bars represent SD. Statistical analysis was performed by Student’s *t* test. *p < 0.05; ***p < 0.001.

In both human beings and mice, a consequence of respiratory viral infections, particularly influenza, is an enhanced susceptibility to secondary bacterial infections (22, 23, 25). To place the OM-85-mediated enhanced antiviral immunity into this highly clinically relevant setting, we treated mice with OM-85 and on day 7 following an influenza infection mice were exposed to a sublethal dose of either *K. pneumoniae* or *S. pneumoniae* (Figure 2A). In the absence of any treatment, the infectious doses of the bacteria that were utilized resulted in mild infections with no weight loss, temperature loss, or signs of morbidity in the bacterial infection only groups, while the dose of influenza resulted in a transient weight loss (data not shown). However, when influenza infected animals were subsequently given the low-dose bacterial infection, bacteremia was present in control treated mice, while pre-treatment with OM-85 protected mice from the bacteremia (Figure 2B). Moreover, OM-85 treatment protected mice from the sequelae resulting from the secondary bacterial infections including weight/temperature loss, and morbidity scores (Figures 2C,D).

### OM-85 enhances dendritic cell maturation

**Figure 3:**
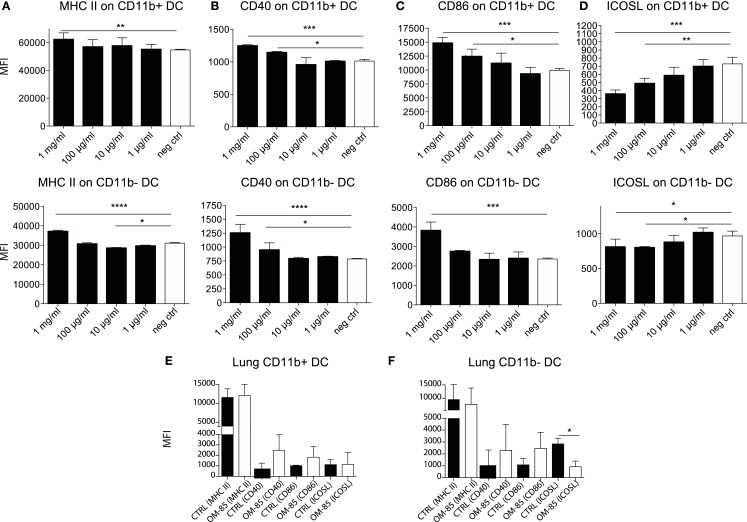
**OM-85 activates dendritic cells *in vivo* and *in vitro***. Splenocytes from naïve mice were activated with the indicated concentration of OM-85 and expression of **(A)** MHC II, **(B)** CD40, **(C)** CD86, or **(D)** ICOSL on CD11b+ and CD11b−. DCs were determined by flow cytometry after 24 h of culture. Mice were treated with OM-85 or control solution for 10 days per oral. Lung cells were then isolated and analyzed by flow cytometry. Surface expression of the indicated markers on **(E)** CD11b+ DC, **(F)** CD11b− DC. Data are representative of 2 experiments. Error bars represent SD. Statistical analysis was performed by one-way ANOVA. *p < 0.05; **p < 0.01; ***p < 0.001; ****p < 0.0001.

Given the protective effect of OM-85 against viral infections and consequently secondary bacterial infections, we next investigated how OM-85 shaped DC activation states, considering that DCs are one of the most potent cell types that influence immunity and have recently been shown to be activated by OM-85 (26). Accordingly, splenocytes were isolated from naïve mice and stimulated with different doses of OM-85 *in vitro*. OM-85 treatment led to a dose-dependent increase in the surface expression of MHC II (Figure 3A), CD40 (Figure 3B), and CD86 (Figure 3C) on both CD11b+ and CD11b− DC populations indicative of an increased activation state. Interestingly, not all markers were upregulated as a clear dose-dependent reduction in costimulatory ligand; ICOSL was found in these same cell populations (Figure 3D). We next investigated whether these markers were regulated *in vivo* and found similar trends in surface marker expression on lung DCs (Figures 3E,F), although no changes were evident on splenic DCs (data not shown), suggesting OM-85 elicited a mucosal tissue-specific effect. Given MHC II and the costimulatory molecules, CD40 and CD86 were increased by OM-85, it is likely that this effect was linked with the observed increase in influenza-specific CD8+ T-cell responses (Figure 1D), although it does not necessarily explain the rapid protection against the virus, which was already apparent in the first days post-infection prior to when the CD8+ T-cell response had developed.

### OM-85 enhances B-cell activation states and production of polyclonal antibodies

**Figure 4:**
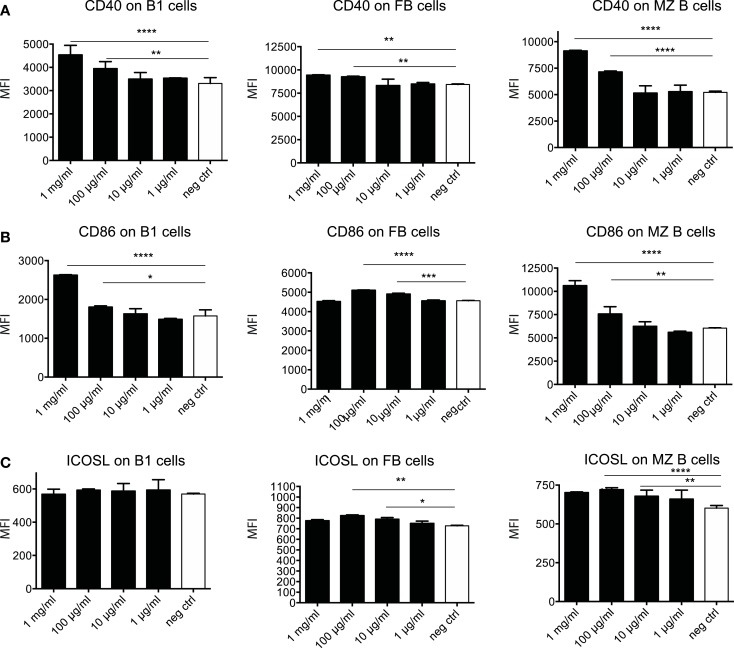
**OM-85 activates B-cells *in vitro***. Splenocytes from naïve mice were activated with the indicated concentration of OM-85 and expression of **(A)** MHC II, **(B)** CD86, or **(C)** ICOSL on B1 cells, follicular B (FB) cells, or marginal zone (MZ) B-cells was determined by flow cytometry after 24 h of culture. Data are representative of 2 experiments. Error bars represent SD. Statistical analysis was performed by performed by one-way ANOVA *p < 0.05; **p < 0.01; ***p < 0.001; ****p < 0.0001.

**Figure 5:**
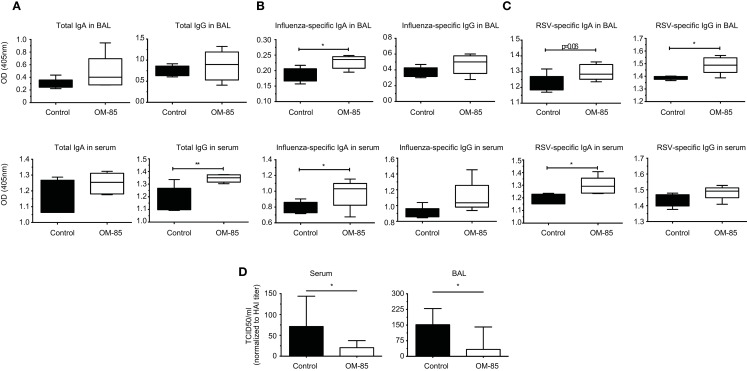
**OM-85 treatment increases polyclonal antibody production with the capacity to neutralize influenza infection**. BALB/c mice were treated with OM-85 or control solution daily for 10 days. The following day serum and BAL fluid were isolated and ELISA performed. **(A)** Total IgA and IgG levels in BAL fluid and serum. **(B)** Influenza antigen specific IgA and IgG in BAL fluid and serum. **(C)** Respiratory syncytial virus specific (RSV) IgA and IgG in BAL fluid and serum. **(D)** Influenza virus was incubated with heat-inactivated serum or BAL fluid from control or OM-85 treated mice, followed by analysis of virus infectivity by TCID50. Data are representative of 2 experiments. Error bars represent maximum to minimum values **(A–C)** or SD **(D)**. Statistical analysis was performed by Student’s *t* test. *p < 0.05; **p < 0.01.

To further dissect the possible mechanism through which OM-85 protects mice against influenza infection and subsequent secondary bacterial infection, we next characterized its effect upon B-cells. In an approach similar to the DC analysis, we stimulated splenocytes with different concentrations of OM-85 and assessed their activation state. Similar to the DC response, CD40 (Figure 4A) and CD86 (Figure 4B) were upregulated in a dose-dependent manner on B1 B-cells, Follicular B (FB) cells and marginal zone (MZ) B-cells. However, contrary to the DCs, these B-cell populations exhibited no significant changes in their expression of ICOSL except, to a minor extent, in MZ B-cells (Figure 4C). In order to determine whether these OM-85 driven changes in B-cell maturation had consequences *in vivo*, we administered OM-85 for 10 days by gavage as performed previously (Figure 1A), and then assessed IgG and IgA in the serum and airways of mice. OM-85 treatment alone led to a statistically significant increase in the levels of IgG detected in the serum of mice and trends toward increased IgA and IgG in the airways (Figure 5A). Of note, however, there was a statistically significant increase in IgA isolated from the serum or airways that bound to influenza antigens (Figure 5B). This was particularly intriguing because the OM-85 treated mice had not been infected with the virus in this setting. Moreover, antibodies that bound respiratory syncytial virus (RSV) were also detectible in the OM-85 treated groups (Figure 5C). These data indicate that OM-85 treatment had led to a polyclonal B-cell activation that resulted in release of antibodies against multiple antigens. Although we could detect comparably higher levels of these antibodies in OM-85 as compared to control treated mice, their relevance was unclear. Thus, to assess whether these polyclonal antibodies had functional implications, we tested their ability to limit influenza virus infection. Indeed, in an *in vitro* virus neutralization assay, heat-inactivated serum or BAL fluid from OM-85 treated mice was effective at limiting influenza infection (Figure 5D). Taken together, these data indicate that OM-85 can act to protect mice against influenza infection by enhancing B-cell activation and release of broadly protective antibodies that help to protect the host against infection.

## Discussion

The underlying risk factors for recurrent RTIs are poorly understood, and the treatment options are limited (27). We hypothesized that one means of addressing this clinical issue would be to provide stimulation to the immune system that might direct it in such a way as to improve mucosal barrier protective pathways. Indeed, some of the most fundamental signals received by cells associated with the mucosa, come from microbes. This host–microbe cross-talk is essential for induction of appropriate immune pathways that limit microbial invasion.

**Figure 6:**
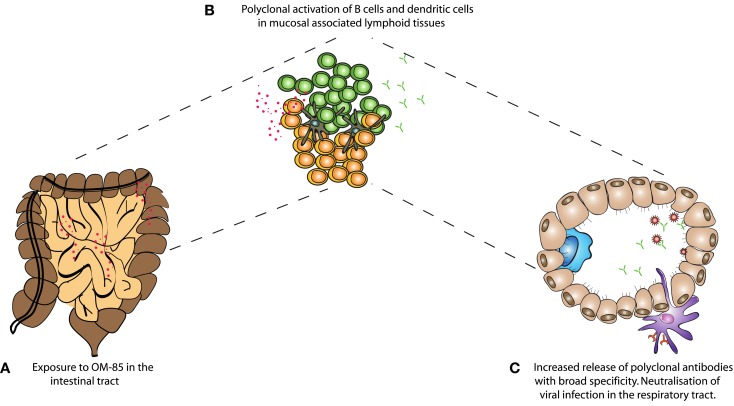
**A schematic representation of the mechanism of action of OM-85 in protection against viral infection**. **(A)** Oral administration of OM-85 leads to **(B)** increased dendritic cell and B-cell activation in the mucosal associated lymphoid tissues. Either by direct activation of cells in the respiratory tract associated lymphoid tissue, or following migration from the gut associated lymphoid tissue, **(C)** OM-85-activated polyclonal B-cells release increased levels of IgA, which are capable of binding and neutralizing influenza virus, protecting against infection and the subsequent susceptibility to secondary bacterial infections.

Overall, we have found that oral administration of a bacterial extract is sufficient to protect animals from a viral infection, as well as the severe consequences of secondary bacterial infections following Influenza. Huber and colleagues have previously shown that oral administration of OM-85 leads to increased levels of IgA in Peyer’s patch and mesenteric lymph node culture supernatants (28). The fact that oral administration of the extract was able to increase IgA and improve mucosal immunity in the airways adds weight to the concept of the “common mucosal immune system”; however, formal mechanistic data showing how intestinal responses influence the respiratory tract are still very limited. It is possible that stimulation of immune cells in the intestinal associated lymphoid tissue results in recirculation of cells to other mucosal sites; alternatively, components of the bacterial extract might reach the circulation and then directly influence cells in the lung, or precursor cells destined for the lung. Considering the absence of surface marker changes observed *in vivo* from spleen cells (data not shown), this is, however, unlikely to be the case. In the current study, it appears that OM-85 was capable of activating a number of different cell types, such as DCs, B–cells, and T-cells. Macrophage phenotypes were also changed albeit with different markers (data not shown). The B-cell stimulation, in particular, could be considered within the context of innate stimulation and the enhancement of a natural non-affinity matured antibody repertoire, capable of binding many different antigens. Indeed, we found that OM-85 treatment resulted in the release of antibodies capable of binding to influenza and RSV, even in the absence of specific exposure to those pathogens. Although these antibodies are unlikely to have an affinity comparable to those having undergone affinity maturation, they were still effective at blocking viral infection. From studies of germ-free mice, it is known that microbial stimulation is critical for the induction of IgA barrier immunity; now our data show oral administration of a bacterial extract alone was capable of improving this fundamental arm of mucosal immunity in the respiratory tract (Figure 6).

In this study, we also assessed the effect of OM-85 upon DC maturation. Perhaps expectedly, stimulating DCs with a bacterial extract resulted in their activation, as noted by increased levels of MHC II, CD40, and CD86. It is intriguing, however, that ICOSL expression was specifically downregulated following OM-85 stimulation. While it is possible that ICOSL follows different kinetics than these other markers, ICOSL–ICOS interactions have particularly been associated with allergic Th2 responses, and OM-85 has been linked with protection against allergic responses (10). Thus, this effect on downregulation of ICOSL expression could indicate one important component of its mechanism of action. Clearly, further studies are warranted in order to dissect the functional consequences of the MHCII^hi^CD86^hi^CD40^hi^ICOSL^lo^ DCs in promoting protective immunity, while enhancing regulatory pathways against allergy.

Taken together, this study highlights the importance of microbial stimulation of the immune system for maintaining effective mucosal immune barriers. We show that oral administration of bacterial products is sufficient to improve systemic and respiratory tract antibody levels, and that the increased presence of these antibodies is associated with enhanced control of primary respiratory viral infection and secondary bacterial infections. These data support the concept of boosting our immune system by providing non-deleterious microbial stimulation for the prevention and treatment of RTIs.

## Conflict of Interest Statement

Christian Pasquali and Jacques Bauer are employees of OM Pharma (Vifor). Manisha Taneja, Olawale Salami, Céline Pattaroni, Aurelien Trompette, Eva S. Gollwitzer, Koshika Yadava, and Benjamin John Marsland declare they have no conflict of interest.
